# Persistence of Peripheral Pulsein Wounds of Arteries

**Published:** 1918-09

**Authors:** 


**Persistence of Peripheral Pulsein Wounds of Arteries,** Fiolle, Le Prog. Med., Feb. 23, noted persistence in 3 of 12 ''dry wounds'' of arterial trunks and advises that the region of the wound should be explored.

